# Creep and flow of glasses: strain response linked to the spatial distribution of dynamical heterogeneities

**DOI:** 10.1038/srep11884

**Published:** 2015-07-08

**Authors:** T. Sentjabrskaja, P. Chaudhuri, M. Hermes, W. C. K. Poon, J. Horbach, S. U. Egelhaaf, M. Laurati

**Affiliations:** 1 Condensed Matter Physics Laboratory, Heinrich Heine University, Universitätsstr. 1, 40225 Düsseldorf, Germany; 2 Theoretical Physics II, Heinrich Heine University, Universitätsstr. 1, 40255 Düsseldorf, Germany; 3 SUPA, School of Physics & Astronomy, The University of Edinburgh, Mayfield Road, Edinburgh EH9 3JZ, United Kingdom

## Abstract

Mechanical properties are of central importance to materials sciences, in particular if they depend on external stimuli. Here we investigate the rheological response of amorphous solids, namely colloidal glasses, to external forces. Using confocal microscopy and computer simulations, we establish a quantitative link between the macroscopic creep response and the microscopic single-particle dynamics. We observe dynamical heterogeneities, namely regions of enhanced mobility, which remain localized in the creep regime, but grow for applied stresses leading to steady flow. These different behaviors are also reflected in the *average* particle dynamics, quantified by the mean squared displacement of the individual particles, and the fraction of active regions. Both microscopic quantities are found to be proportional to the macroscopic strain, despite the non-equilibrium and non-linear conditions during creep and the transient regime prior to steady flow.

The properties of materials not only depend on their chemical composition, but also on the arrangement and dynamics of their constituents. It is thus crucial to understand the link between the macroscopic behaviour and the microscopic single-particle level. The relation between an applied mechanical force and microscopic processes is understood for crystalline, i.e. ordered, materials. Crystalline solids (like metals, ceramics or minerals) irreversibly deform when subjected to a load which is small enough to avoid fracture, although this response is very slow. This kind of response is called creep and originates from the presence of defects in the otherwise ordered arrangement of atoms. The diffusion of vacancies and dislocations is responsible for the observed plastic deformation^1^. The same relation and microscopic processes cannot occur in amorphous, i.e. disordered, materials.

Nevertheless, in amorphous solid-like materials, a similar *macroscopic* creep response is observed under application of shear stresses below the yield stress *σ*_y_ , i.e. below the transition from an elastic to a plastic response. The macroscopic creep response has been intensively studied in metallic, polymeric and colloidal glasses^2^,^3^,^4^,^5^,^6^,^7^. Several models^8^,^9^,^10^,^11^,^12^,^13^,^14^,^15^, successfully describe the time evolution of the strain measured during creep, namely its characteristic sub-linear time dependence. However, the relation of the creep response to the microscopic structure and dynamics has hardly been determined and is not well understood. Due to the disordered structure of amorphous solids the concept of defects is not applicable and a microscopic mechanism different from the one in crystalline solids must be responsible for creep. Thus, to make progress, microscopic observations on a single-particle level during creep tests are required.

Combining experiments and simulations, we investigated colloidal glasses when constant stress is suddenly applied, i.e. during creep tests . We reveal a quantitative link between the macroscopic rheological response and the microscopic dynamics. This is possible due to recent developments in simultaneously performing rheology and confocal microscopy^16^,^17^. During creep flow near the yielding threshold, we observe that very few particles undergo large non-affine displacements which leads to pronounced, but spatially localized, dynamical heterogeneities and sub-diffusive dynamics. In contrast, for stresses beyond the yield stress, transient super-diffusive dynamics mark the onset of steady flow. At the same time, growing domains of enhanced dynamic activity are present, with their number correlating with the macroscopic strain. This is reflected in a correlation between the macroscopic strain and the single particle displacements. In addition to the steady-state flow regime, this correlation also holds in the creep and transient states, specially for stresses near the yield stress. Hence, we can quantitatively relate the macroscopic rheological response of soft glasses to the average and heterogeneous microscopic dynamics which are spatially localized during creep but span the entire system at large stresses that lead to flow. The different microscopic behavior thus reflects the different macroscopic response during creep and flow, respectively. This extends previous observations to non-linear and non-equilibrium situations. Furthermore, as we observe the same behavior for different systems, realized in the experiments and simulations, this appears to be a general feature of glasses.

## Results and Discussion

In our experiments and simulations we investigated two different model colloidal glasses. In the experiments, the glass is a binary mixture of sterically stabilised PMMA spheres with a size ratio of 5, dispersed in a density and refractive index matching solvent, with total volume fraction *ϕ* = 0.61 and a relative volume fraction of small spheres *x*_S_ = *ϕ*_S_/*ϕ = *0.1. In this binary glass, the motion of the large particles is arrested via caging by neighbouring large particles^18^,^19^,^20^. In our molecular dynamics simulations, the glass is formed by a binary Yukawa fluid of large and small spheres with size ratio 1.2 and a relative number fraction of small spheres of 0.5, large enough to prevent crystallization. This system is quenched to *T* = 0.10, i.e well below the mode-coupling critical temperature of the system, *T*_c_ = 0.14. All times are normalized; in the experiments by the short-time one-dimensional Brownian diffusion time of the large spheres, 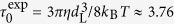 s, where *d*_L_ is the diameter of the large spheres , *η* is the viscosity and *k*_B_*T* the thermal energy, and in the simulations by the time unit 
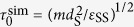
, where *m*, *d*_S_ and 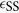 are units of mass, length and energy, respectively, with *d*_S_ the diameter of the small spheres and 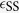 the energy-scale corresponding to the interaction between small particles. The colloidal glasses investigated in experiments and simulations hence involve different interactions and different mixing and size ratios of their components. Using these different model systems allows us to explore the general features in the response of glasses to externally applied stresses.

### Macroscopic Strain is Quantitatively Related to Single-Particle Mean Squared Displacement

We performed a step to an applied constant stress (*σ *=* *const) on an initially quiescent glass. In the experiments, the stress was applied using a commercial stress-controlled rheometer, while in the simulations one wall was pulled by a constant force *F*_0_. We monitored the macroscopic response via the time evolution of strain *γ*(*t*). This situation is in contrast to the case of imposing a constant shear rate (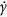
* *=* *const)^21^,^22^,^23^,^24^,^25^,^26^,^27^, where the bulk stress *σ*(*t*) is monitored. Unlike for an applied shear rate 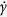, when constant stress *σ* is applied there is no timescale imposed and flow regimes below yielding can be investigated. The choice of control parameter, i.e. constant *σ* or constant 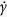, hence determines the intermediate flow states via which a glass evolves from the quiescent state to steady flow^11^. In the following, we exploit these possibilities and link the increasing macroscopic strain to the evolution of local particle motions, using stress as the external variable and including stresses below the yield stress *σ*_y_ . In experiments, we estimated the yield stress of the glass, *σ*_y_ ≈ 0.010 Pa, from the stress at the crossing point of the storage and loss moduli in large amplitude oscillatory shear measurements at 1 rad/s. In simulations, at *T* = 0.10 the yield stress *σ*_y_ = 0.072 (in simulation units) was estimated by strain-rate controlled simulations^28^.

**Figure 1:**
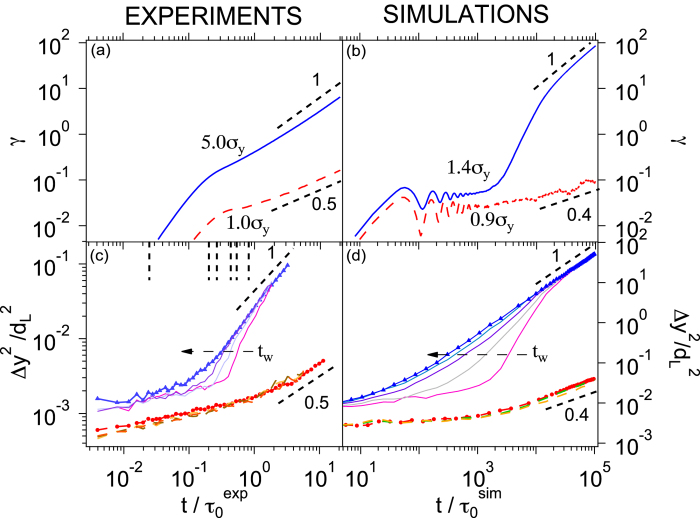
Comparison of (left) experimental and (right) simulation results. (top) Time-dependence of the strain *γ*(*t*) for applied stresses *σ* as indicated, relative to the yield stress *σ*_y_. (bottom) Mean squared displacement in the vorticity direction, Δ*y*^2^, (indicated by same colors and line styles), immediately after stress application, i.e. for waiting time *t*_w_ = 0, and larger *t*_w_ (as indicated) until the steady-state is reached, i.e. *t*_w_→∞ (symbols). For the smaller applied stress, Δ*y*^2^ is divided by a factor 3 for clarity, both in experiments and simulations.

If the applied stress *σ* ≈ *σ*_y_ , a characteristic creep response is observed with the strain increasing sub-linearly with time within the experimental window, γ ~ *t*^*a*^ with *a* ≈ 0.5 (Fig. 1a, broken line). Furthermore, for *σ* = 0.9*σ*_y_ (Fig. 1b, broken line), a smaller effective exponent is found, in agreement with previous results^6^,^12^,^28^,^29^,^30^. Hence, for 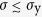 , the deformation occurs extremely slowly and the system is unable to reach a steady state within the observation time. This is reflected in the particle dynamics in vorticity (neutral) direction, namely the mean squared displacement (MSD) Δ*y*^2^(*t*) (Fig. 1c,d, broken lines). In experiments and simulations, at short times the increase of the MSDs is limited, consistent with caging, while at longer times a sub-diffusive regime is observed; Δ*y*^2^ ~ *t*^*b*^ with *b *<* *1. We find *b* ≈ *a* within the explored time window. The MSDs show little change with the waiting times *t*_w_ after the beginning of the stress application (Fig. 1c,d, broken lines). The observed macroscopic creep response and the absence of steady-state flow is thus connected to the particles’ inability to diffuse.

In contrast, if 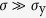 , the strain response shows a rapid transition to a steady flow regime, which corresponds to *γ *~ *t*, i.e. 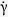 is constant (Fig. 1a,b, solid lines). The MSDs again display caging at intermediate times (Fig. 1c,d, solid line), while at long times diffusion^27^,^31^,^32^,^33^. The slightly lower MSD plateau observed in experiments is due to cage constriction^18^, and is also observed in Brownian dynamics simulations^24^,^27^, but not in molecular dynamics simulations where the microscopic dynamics is Newtonian. In between caging and long time diffusion, a transient super-diffusive regime is observed. This coincides with the transition of the rheological response from the initial elastic regime to the flow regime. Note that in the experiments, the initial superlinear increase in strain is a known effect of the rheometer’s inertia^34^. With increasing waiting time *t*_w_ (Fig. 1c,d), super-diffusion occurs at increasingly earlier times and for increasingly shorter time intervals, until it almost disappears in the steady state. Thus, the onset of flow is characterized by transient super-diffusion and, subsequently in the steady state, by diffusion. This indicates that the different regimes in the macroscopic strain response *γ*(*t*) are reflected in different features of the single-particle dynamics, here characterized by the MSD Δ*y*^2^(*t*).

**Figure 2:**
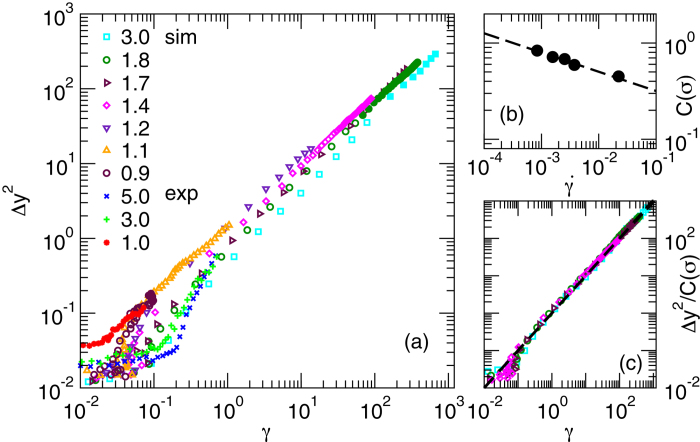
(**a**) Mean squared displacement in vorticity direction, Δ*y*^2^, as a function of strain *γ* for different values of the applied stress *σ*/*σ*_y_ obtained in experiments and simulations (Sec. 1 of Supplementary Information). The experimental Δ*y*^2^ values are multiplied by a constant factor in order to match the simulation data. (**b**) Ratio of diffusion coefficient to shear rate, 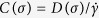, obtained from fits (Sec. 1 of Supplementary Information), as a function of the shear rate in the steady state, 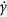. The dashed line indicates a power-law 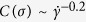. (**c**) Scaling plot of Δ*y*^2^/*C*(*σ*) as a function of *γ*, with the dashed line indicating a slope of 1.

We now quantitatively investigate the relation between the macroscopic strain *γ*(*t*) and the microscopic MSDs Δ*y*^2^(*t*). In the case of steady flow 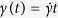 and, since then the particles diffuse, Δ*y*^2^(*t*) ~ *D*(*σ*)*t*, which implies that 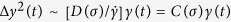. Previous experiments and simulations under constant applied shear rate have found 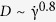 21,22, which implies 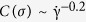, since stress and strain control are equivalent in steady flow. In our case, in the asymptotic diffusive regime (corresponding to 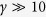, Fig. 1a,b) we observe an approximate linear relation Δ*y*^2^(*t*) ~ γ(*t*) for a large range of *σ* (Fig. 2a, Sec. 1 in Supplementary Information). The slight shifts between the curves for different *σ* occur due to the expected behaviour of *C*(*σ*) (Fig. 2b). If Δ*y*^2^ is rescaled by *C*(*σ*), the data fall onto a single line of slope 1 (Fig. 2c).

Although our argument for the relation Δ*y*^2^(*t*) ~ *γ*(*t*) is based on the assumption of steady flow, the relation surprisingly also holds in non-steady states for 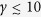, which corresponds to creep (for 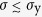) or the transient regime before steady flow (for *σ* > *σ*_y_). In contrast, Δ*y*^2^(*t*) ~ *γ*(*t*) does not hold for large stresses *σ* and small strains *γ* (or short times *t*). In both, experiments and simulations, systematic deviations are seen to occur with increasing stress. The deviations occur due to a time lag between the particles’ motion beyond their cages and the onset of macroscopic deformation (Fig. SM-1, Supplementary Information). Moreover, at very short times, i.e. in the initial elastic regime (Fig. 1), the proportionality is also not observed. This suggests that the observed correlation is a consequence of the plasticity that develops after the initial elastic regime. Our observations mark a clear difference between the yielding response under applied constant stress (investigated here) and applied constant shear rate (investigated in^24^,^26^). In the latter, Δ*y*^2^(*t*) ~ *γ*(*t*) cannot hold in the transient regime, where Δ*y*^2^(*t*) increases superlinearly with *t* while *γ*(*t*) increases linearly. Note that this connection between nonlinear strain and the single-particle dynamics is an implicit assumption in a recent theoretical approach based on a nonlinear Langevin equation^35^,^36^,^37^. Our data indicates to what extent such a connection is valid.

### Displacement Distributions Indicate Small Fraction of Mobile Particles

**Figure 3:**
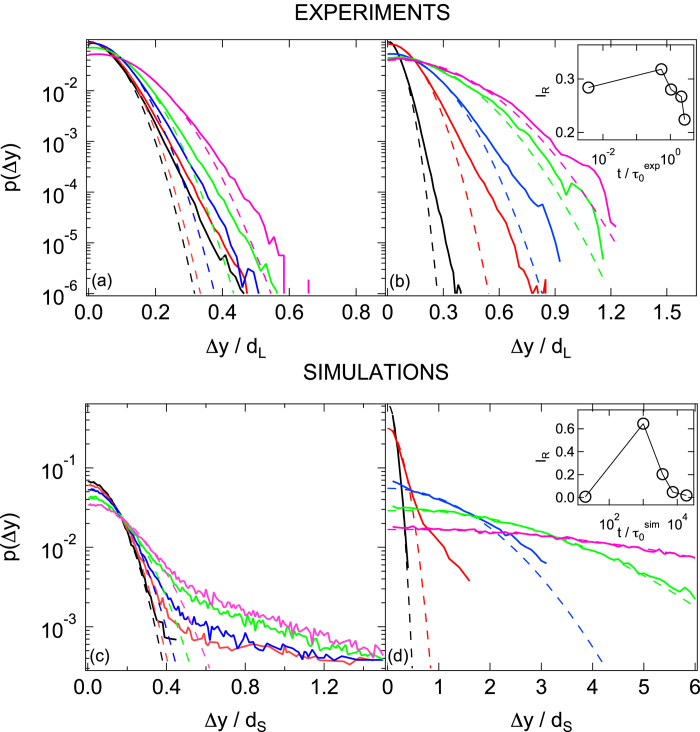
Van Hove self-correlation functions, i.e. distributions of displacements *p*(Δ*y*), determined (top) by experiments for (**a**) a stress *σ* ≈ *σ*_y_, a waiting time *t*_w_ = 0 and times 
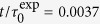
, 0.54, 1.05, 2.12 and 5.31 (left to right) and (**b**) *σ* ≈ 5*σ*_y_, *t*_w_ = 0 and same times, except the longest time here is 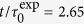, (bottom) by simulations for (**c**) *σ* ≈ 1.1*σ*_y_, *t*_w_ = 0 and 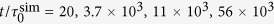 and 110 × 10^3^ (left to right) and (**d**) *σ* ≈ 1.53*σ*_y_, *t*_w_ = 0 and 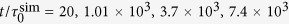 and 18.6 × 10^3^ (left to right). Dashed lines represent Gaussian fits to *p*(Δ*y*) for small Δ*y*. Insets: normalized integral *I*_R_ of the residuals of the Gaussian fits in the main plots, as a function of time *t*/*τ*_0_.

In addition to the characterisation of the particle displacements via the MSD, i.e. a mean value, we have also investigated the distribution of the displacements, namely the self part of the van Hove function *p*(Δ*y*). For *σ* ≈ *σ*_y_ (Fig. 3, left), at all times the van Hove functions exhibit a nearly Gaussian shape for small Δ*y*, which corresponds to localised particles, and moderate exponential tails which correspond to large displacements of a small fraction of particles. The non-Gaussian tails only slightly change with increasing time. This indicates that shear-induced delocalisation is a very slow process. In particular, large displacements at the shortest time of the measurement 
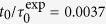
 hardly occur and therefore macroscopic flow is delayed.

For *σ* > *σ*_y_ (Fig. 3, right) shear leads to a larger deviation from a Gaussian distribution with a significant number of large displacements. The deviation from Gaussian behavior was quantified by the time dependence of the integral *I*_R_(*t*) of the residuals of the Gaussian fits to *p*(Δ*y*), which, for each fixed time, was normalised to the integral of the distribution (Fig. 3, inset). A non monotonic trend of *I*_R_(*t*) is observed, with a maximum value during the intermediate super-diffusive regime. At later times *I*_R_(*t*) continuously decreases and eventually vanishes when diffusion sets in and a Gaussian distribution of displacements is recovered.

### Evolution of Dynamical Activity Follows Macroscopic Strain

**Figure 4:**
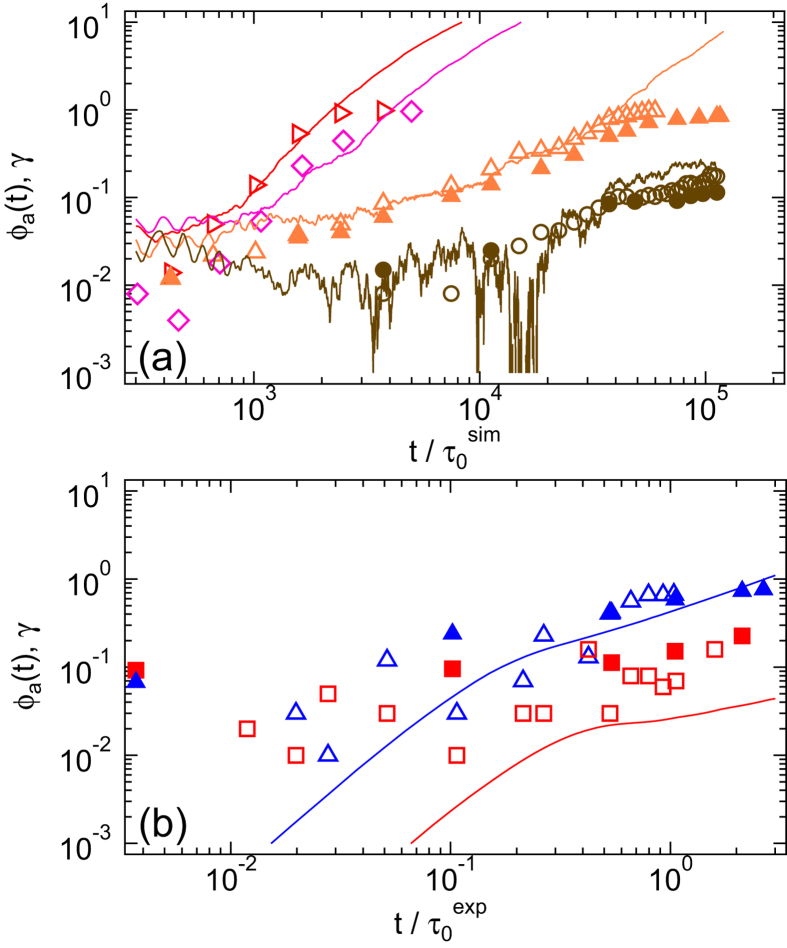
Fraction of active particles 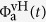 (solid symbols) and active boxes 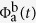 (open symbols) determined (**a**) by simulations at stresses *σ*/*σ*_y_ = 0.9 (●, ○), 1.1 (▲, ∆), 1.4 (◊), 1.53 (▷) and (**b**) by experiments at *σ*/*σ*_y_ ≈ 1.0 (■, □) and 5.0 (▲, ∆). Lines of the same colour represent the strain γ for the corresponding applied stresses, where the instantaneous strain is shown in the case of the simulations.

The tails in the van Hove function *p*(Δ*y*) reveal the existence of a small number of very mobile particles during the transient regime. We quantify the time evolution of the fraction of these mobile particles by the ratio 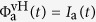 with *I*_a_ the integral of *p*(Δ*y*) for displacements Δ*y*/*d* > 5Δ*y*_min_, with *d* = *d*_S_ and *d*_L_ in simulations and experiments, respectively (Fig. 4, solid symbols). The value 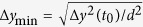 is the localization length estimated from the MSDs at the shortest time *t*_0_ (Fig. 1c,d). In simulations the time-dependence of 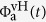 closely follows that of the instantaneous strain *γ*(*t*), up to 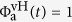 (Fig. 4a, lines). In the experiments, similar results for 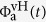 are observed (Fig. 4b) except that, in contrast to the simulations, *γ*(*t*) is not the instantaneous strain but a time average, leading to a small deviation between 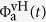 and *γ*(*t*). The macroscopic strain is therefore not only proportional to the average mean squared displacement (Fig. 2) but also the fraction of mobile particles: this indicates that the mobile, dynamically active particles contribute most significantly to the mean squared displacement. This is true both below and above the yield stress.

### Spatial Distribution of Dynamical Activity is Heterogeneous

We introduce spatial coarse-graining in order to reduce noise. We divide the field of view into 10 × 10 square boxes, each with size (2.8 *d*_L_)^2^. For each particle *i*, the displacement in the vorticity direction, Δ*y*_*i*_(*t*) = *y*_*i*_(*t*)–*y*_*i*_(*t*_0_), was determined. The average particle mobility in box *lm*, with *l*, 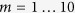, was calculated according to


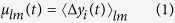


where 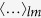 denotes an average over all the particles which were in the box *lm* at *t* = *t*_0_. A box *lm* is defined active at time *t* if *μ*_*lm*_ > 5Δ*y*_min_, following the same criterion used to distinguish largest displacements of single particles in the van Hove functions (Fig. 1c,d). The fraction of active boxes, 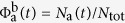, with *N*_a_ the number of active boxes and *N*_tot_ the total number of boxes. With time 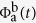 grows as the fraction of the single mobile particles 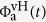 (Fig. 4, symbols). Thus, the time-dependence of 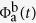 is also proportional to γ(*t*). A similar connection between the number of active regions and strain growth was experimentally observed in the creep flow of frictional granular particles^38^.

**Figure 5:**
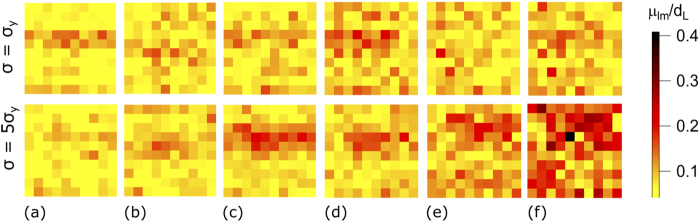
Maps of average particle mobilities *μ*_*lm*_(*t*) within boxes *lm* (Eq. (1)) for (top) stress *σ* ≈ *σ*_y_ and (bottom) *σ* ≈ 5*σ*_y_ and times 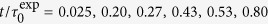 (**a**–**f**, indicated in Fig. 1c by dashed lines) as observed in experiments. The box size is (2.8 *d*_L_)^2^.

To investigate the existence of heterogeneity in the dynamical activity, we consider the spatial distribution of active boxes. For *σ* ≈ *σ*_y_ , the distribution of local mobilities within the velocity-vorticity plane does not indicate any prominent features (Fig. 5, top). At any specific time, there are some active boxes with larger mobilities, but the locations of the boxes with the largest mobilities vary randomly with time. For *σ* ≈ 5*σ*_y_, similar mobilities occur at short times, when the localisation plateau in the MSD is observed (Fig. 5a,b, bottom). In contrast, at 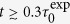, roughly coincident with the onset of super-diffusion in the MSDs determined for *t*_w_ = 0 (Fig. 1c), a region with enhanced mobilities emerges (Fig. 5c,d, bottom), expands with time (Fig. 5e, bottom) and spans almost the whole field of view once the system flows (Fig. 5f, bottom). Hence, the onset of flow (Fig. 1a,b) coincides with the appearance of a region of higher local mobility (Fig. 5) and super-diffusive dynamics (Fig. 1c,d). Furthermore, it leads to the pronounced non-Gaussian tails in the van Hove correlation function at intermediate times (Fig. 3), which disappear once steady flow has developed and the dynamics again becomes more homogeneous (Fig. 3, inset).

The enhanced local mobilities do not result from sudden large displacements, but occur through the accumulation of only slightly above-average local, non-affine particle displacements. This has been confirmed by calculating the instantaneous mobilities from 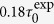 to 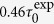 and 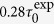 to 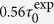, i.e. for 10 sampling times, instead of starting from the shortest measurement time (as in Fig. 5). No large instantaneous mobilities and no significant difference to *σ* ≈ *σ*_y_ are observed (Sec. 2 in Supplementary Information). Similar results are obtained in our simulations. The occurrence of correlated plastic events^39^,^40^ and avalanche-like behavior^41^,^42^ have been proposed as mechanisms driving the onset of flow. Such cooperative events might be connected to the correlated local mobilities and their spreading observed in our study. The observed intermittency in the displacements might also be related to stick-slip motion^43^.

**Figure 6:**
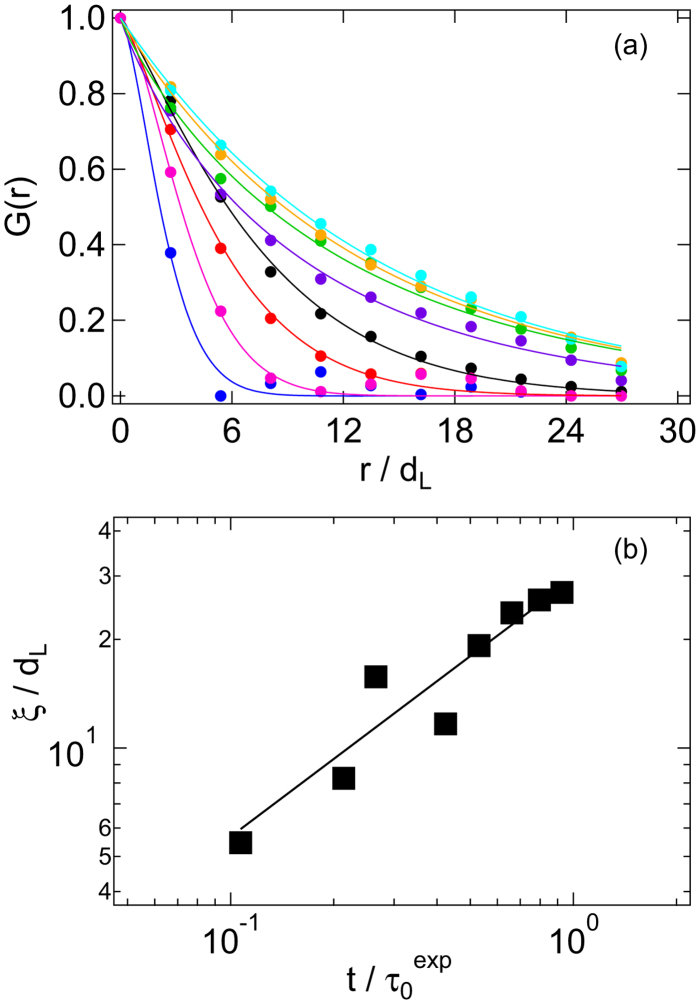
(**a**) Box-box correlation functions *G*(*r*) for stress *σ/σ*_y_ ≈ 5 and time 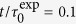, 0.20, 0.27, 0.43, 0.53, 0.66, 0.80 and 0.93 (left to right) as observed in experiments. Lines represent stretched exponential fits. (**b**) Correlation length of active boxes, *ξ*, as a function of time for *σ/σ*_y_ ≈ 5.0; the line indicates *ξ*/*d*_L_ ~ *t*^2/3^.

The larger area in the velocity-vorticity plane monitored in the experiments allows us to quantitatively investigate the spatial growth of active regions. If the box *lm* is active or inactive, *n*_*lm*_ is defined as 1 or 0, respectively. Based on this definition, we calculate the spatial correlation of active boxes, that is the box-box correlation function, 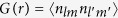 with *r*^2^ = (*l*–*l*′)^2^ + (*m*–*m*′)^2^ (Fig. 6a). The brackets 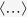 indicate an average over the individual boxes. The characteristic length *ξ* of the spatial correlation *G*(*r*) was determined by fitting a stretched exponential function 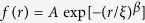 to *G*(*r*). The correlation length *ξ*(*t*) increases from an initial value *ξ* ≈ 5*d*_L_ at 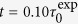 to *ξ* ≈ 30*d*_L_ at 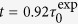, with *ξ*(*t*) ~ *t*^2/3^ (Fig. 6b). For *σ* ≈ *σ*_y_ the correlation length *ξ*(*t*) instead does not grow and stays approximately constant for all times *t* (data not shown).

## Conclusions

Using experiments and simulations, we demonstrated that under applied stress, the macroscopic deformation of glasses can be linked in a consistent way to the single particle displacements. In particular, the strain is approximately linearly related to the single-particle MSD even in the time-dependent non-linear response regime, including the creep and the transient regime preceding steady flow. Furthermore, the fraction of active particles in the van Hove function as well as the fraction of active regions, i.e. of groups of particles, is also proportional to the macroscopic strain. Heterogeneities in the location of these active particles are present both for applied stresses smaller and larger than the yield stress. The spatial distribution of regions with larger displacements determines the onset of flow. For applied stresses around the yield stress, i.e. during creep, localised regions of enhanced dynamical activity allow only for sub-diffusive dynamics. Increasing the stress beyond the yield stress, the active regions grow heterogeneously and super-diffusive transients emerge leading to particle diffusion with steady flow setting in. We observe qualitatively the same behavior for the different models studied in our experiments and simulations and thus expect that our observations represent generic features of glasses.

Future work should focus on understanding how the external stress leads to the occurrence of locally enhanced mobilities, e.g. whether these are related to thermally activated local structural changes. Furthermore, the mechanisms that drive the spreading of the active regions within the plane as well as in the transverse direction need to be identified, thereby providing possible links to transient shear banding in the velocity-gradient plane^28^,^44^. All these would help to develop a more complete scenario for the fluidisation of glassy systems under applied stress. Furthermore, it can open the route to the rational design of materials with desired response to applied stresses.

## Methods

### Experiments

We investigated a mixture of sterically stabilized PMMA spheres of diameters *d*_L_ = 1.76 *μ*m (fluorescently labeled) and *d*_S_ = 0.36 *μ*m, dispersed in a cis-decalin/cycloheptyl-bromide mixture which closely matches their density and refractive index. After addition of salt (tetrabutylammoniumchloride), this system presents hard-sphere like interactions^45^,^46^. The total volume fraction is *ϕ* = 0.61 and the relative fraction of small spheres *x*_S_ = *ϕ*_S_/*ϕ* = 0.1. The formation of a glassy state in this mixture was demonstrated by using rheology and confocal microscopy measurements of the dynamics of large particles^18^,^19^,^20^. The presence of small spheres, with their larger energy density, increases the yield stress of the system, thereby improving the quality of the rheological data while still allowing for the simultaneous observation of the large spheres with confocal microscopy^16^,^17^. The rheological and confocal microscopy measurements reported in the manuscript were obtained using a combination of a commercial MCR-301 WSP stress-controlled rheometer (Anton-Paar) and a VT-Eye confocal unit (Visitech) mounted on a Nikon Ti-U inverted microscope, with a Nikon Plan Apo 60x oil immersion objective (NA = 1.40). We used a cone-plate geometry of diameter 50 mm, cone angle 1° and truncation gap 100 *μ*m. The bottom plate consists of a microscope coverslip which was coated with a mixture of PMMA particles of radius 0.885 *μ*m and 0.174 *μ*m. The surface of the cone is sandblasted. The roughness of the geometries prevents wall-slip, as verified by imaging. A solvent trap was used to reduce solvent evaporation. Due to the fact that rheological measurements on colloidal glasses are affected by loading effects, shear history and aging, before each test a renjuvenation procedure was performed in order to obtain a reproducible initial state of the system. After loading, we performed a dynamic strain sweep to estimate the yield strain *γ*_y_ of the system from the crossing point of the strain-dependent storage, G′, and loss, G′′, moduli. Oscillatory shear at 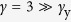 was applied to induce flow and maintained until the time-dependent G′ and G′′ reached a stationary state, typically after 200 s. Afterwards, oscillatory shear in the linear viscoelastic regime, *γ* = 0.001, was applied until G′ and G′′ became stationary, typically for *t* > 300 *s*. The state characterised by the stationary values of G′ and G′′ was the initial state, prepared before each creep measurement. The relative error on the strain determination during creep measurements is smaller than 1%.

Confocal microscopy images were acquired in a velocity-vorticity plane about 6 mm from the center of the geometries and 30 *μ*m from the bottom plate. Images with 512 × 512 pixels, corresponding to 51 *μ*m × 51 *μ*m, were acquired at a rate of 67 frames per second, which ensured accurate particle tracking even at the highest applied stresses (typical movies in Supplementary Information). By imaging the truncation gap of the cone, we verified that bending of the coverslip is negligible^16^. This is also indicated by the fact that, despite the applied stress, the particles in the imaging plane remain perfectly in focus (movies in Supplementary Information). The fact that we can image the truncation gap of the cone is also used to check that the bottom plate is perpendicular to the rotation axis of the cone. Particle coordinates and trajectories were extracted from the images using standard routines^47^. Mean squared displacements from four independent measurements were averaged. The noise contribution to our MSD data was estimated from the MSD of an immobile sample, resulting in 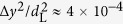, i.e. a factor of about 2.5 times smaller than the Δ*y*^2^(*t*) values measured at short times.

### Simulations

In our molecular dynamics simulations, a 50:50 binary Yukawa fluid of large and small spheres with size ratio 1.2 is investigated. The model parameters have been reported earlier^26^,^28^,^48^. Our simulations have been performed for samples consisting of *N* = 12800 particles and having dimensions *L*_x_ = 26.66*d*_S_, *L*_y_ = 13.33*d*_S_, *L*_z_ = 53.31*d*_S_. We work in the *NVT* ensemble using periodic boundary conditions, the temperature being controlled by a Lowe thermostat^49^. The mode-coupling critical temperature of the system is *T*_*c*_ = 0.14. The system is equilibrated at *T* = 0.15 and then instantaneously quenched to *T* = 0.10, where it is aged for 10^4^ 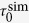. At this time, the walls are generated by freezing particles at 0 < z < 2*d*_S_ and *L*_z_–2*d*_S_ < *z* < *L*_z_ 28. Stress is applied by pulling one wall at a constant force *F*_0_ in the *x* direction. For each applied stress, runs over 24 independent replicas of the system were averaged. Similar to the experiments, the dynamics were measured in a slice at the centre of the volume having thickness 13.3*d*_*S*_ and distance about 18*d*_*S*_ to the walls on each side.

## Additional Information

**How to cite this article**: Sentjabrskaja, T. *et al.* Creep and flow of glasses: strain response linked to the spatial distribution of dynamical heterogeneities. *Sci. Rep.* **5**, 11884; doi: 10.1038/srep11884 (2015).

## Supplementary Material

Supplementary Information

Supplementary Information

Supplementary Information
