# Supplementary material for: Creep and flow of glasses: strain response linked to the spatial distribution of dynamical heterogeneities
Source: Sci Rep. 2015 Jul 8;5:11884. doi: 10.1038/srep11884 (PMC4495392; doi:10.1038/srep11884)
Supplement: Supplementary Information [file srep11884-s3.pdf]

Supplemental Material for 'Creep and flow of glasses: strain response linked to the spatial distribution of dynamical heterogeneities'

T. Sentjabrskaja[1], P. Chaudhuri[2], M. Hermes[3], W. C. K. Poon[3], J. Horbach[2], S. U. Egelhaaf[1], and M. Laurati[1]

[1] *Condensed Matter Physics Laboratory, Heinrich-Heine University, Universitätsstr. 1, 40225 Düsseldorf, Germany.*

[2] *Theoretical Physics II, Heinrich-Heine University, Universitätsstr. 1, 40255 Düsseldorf, Germany.*

[3] *SUPA, School of Physics & Astronomy, The University of Edinburgh, Mayfield Road, Edinburgh EH9 3JZ, United Kingdom.*

In the first section we report the time evolution of the strain $\gamma(t)$ for further values of the applied stress σ, and the corresponding transient mean squared displacements for waiting time $t_w = 0$, complementing the data of Fig. 1 in the main manuscript. In the second section, maps of instantaneous experimental particle mobilities are shown for comparison with the average mobilities.

I. STRAIN EVOLUTION AND TRANSIENT MEAN SQUARED DISPLACEMENTS FOR ADDITIONAL APPLIED STRESSES

The time evolution of the strain $\gamma(t)$ is shown for additional values of the applied stress σ (Fig. SM-1), complementing the data presented in Fig. 1a,b of the manuscript. The corresponding mean squared displacements $\Delta y^2(t)$, determined immediately after application of stress, i.e. with waiting time $t_w = 0$, are also shown (lower panels). The data of Fig. 1 are also reported for comparison. The transition from a creep to a flow response occurs with increasing σ and is found to be gradual. The simulations show that the super-linear regime of the stress and the super-diffusion, both observed for $\sigma \gtrsim \sigma_y$, are especially pronounced for $\sigma \approx \sigma_y$, while they become less pronounced for larger stresses.

For the imposed stresses, when diffusion is observed at long times, we can determine the corresponding diffusion constants $D(\sigma)$ from the mean squared displacement $\Delta y^2(t)$. Similarly, the steady state strain rate $\dot{\gamma}$ can be obtained from the corresponding long-time data for strain $\gamma(t)$. Thus, $C(\sigma) = D(\sigma)/\dot{\gamma}$, defined in the main text, can be calculated and yields $C(\sigma) \sim \dot{\gamma}^{-0.2}$ (Fig. 2b). Taking this into account, i.e. by plotting $\Delta y^2/C(\sigma)$ as a function of strain γ, we obtain a collapse of the data, as shown in Fig. 2c.

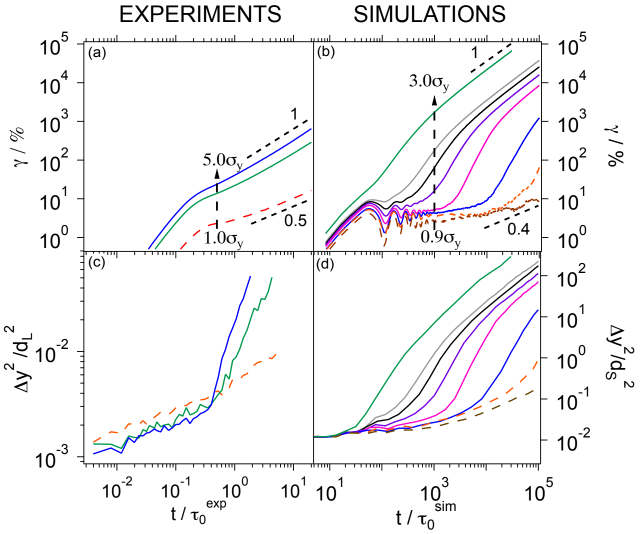

Fig-SM. 1. Comparison of (left) experimental and (right) simulation results. (top) Time-dependence of the strain $\gamma(t)$ for applied stresses (a) $\sigma/\sigma_y \approx 1.0$, 3.0 and 5.0, and (b) $\sigma/\sigma_y = 0.90, 1.10, 1.18, 1.39, 1.53, 1.67,$ 1.80 and 3.0 (bottom to top). (bottom) Mean squared displacement in the vorticity direction, $\Delta y^2(t)$, for the same applied stresses (indicated by the same colors and line styles) immediately after stress application, i.e. for waiting time $t_w = 0$.

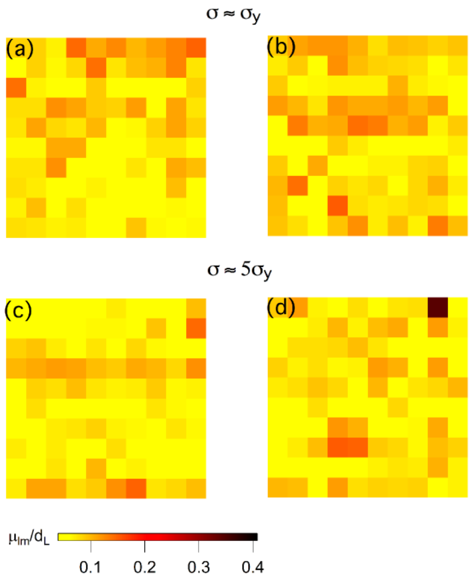

Fig-SM. 2. Maps of instantaneous experimental particle mobilities $\mu_{lm}(t)$ for $\sigma/\sigma_y \approx 1$ (top) and $\sigma/\sigma_y \approx 5$ (bottom), from (a,c) $0.18\tau_0^{\rm exp}$ to $0.46\tau_0^{\rm exp}$ and (b,d) $0.28\tau_0^{\rm exp}$ to $0.56\tau_0^{\rm exp}$. Each box has size $(2.8d_L)^2$.

II. MAPS OF INSTANTANEOUS DISPLACEMENTS

Instantaneous mobilities were calculated from $0.18\tau_0^{\rm exp}$ to $0.46\tau_0^{\rm exp}$ and $0.28\tau_0^{\rm exp}$ to $0.56\tau_0^{\rm exp}$, for $\sigma/\sigma_{\rm y} = 1.0$ (Fig. SM-2a,b) and $\sigma/\sigma_{\rm y} = 5.0$ (Fig. SM-2c,d). No regions of large instantaneous and correlated mobilities are observed. Moreover, the maps for $\sigma/\sigma_{\rm y} \approx 1$ and $\sigma/\sigma_{\rm y} \approx 5$ are comparable, contrary to what was observed in the average mobility maps (Fig. 4). This indicates that the enhanced average mobilities observed in Fig. 4 are not the result of sudden large displacements but rather occur through the accumulation of slightly above-average displacements of particles in a specific region which seem to occur with a slightly larger probability in the beginning (compare Fig. SM-2c,d to Fig. 4c).

III. MOVIES OF SHEARED SUSPENSIONS

Typical movies corresponding to series of confocal microscopy images of the sheared glass, for $\sigma \approx \sigma_{\rm y}$ and $\sigma \approx 5\sigma_{\rm y}$, acquired in a velocity-vorticity plane about 6 mm from the center of the geometries and 30 μm from the bottom plate. Images with 512×512 pixels, corresponding to 51 μm × 51 μm, were acquired at a rate of 67 frames per second.
